# Monitoring Tumor Hypoxia Using ^18^F-FMISO PET and Pharmacokinetics Modeling after Photodynamic Therapy

**DOI:** 10.1038/srep31551

**Published:** 2016-08-22

**Authors:** Xiao Tong, Avinash Srivatsan, Orit Jacobson, Yu Wang, Zhantong Wang, Xiangyu Yang, Gang Niu, Dale O. Kiesewetter, Hairong Zheng, Xiaoyuan Chen

**Affiliations:** 1 Laboratory of Molecular Imaging and Nanomedicine, National Institute of Biomedical Imaging and Bioengineering, National Institutes of Health, Bethesda, Maryland 20892, United States; 2 Paul C. Lauterbur Research Center for Biomedical Imaging, Institute of Biomedical and Health Engineering, Shenzhen Institutes of Advanced Technology, Chinese Academy of Sciences, Shenzhen 518055, China

## Abstract

Photodynamic therapy (PDT) is an efficacious treatment for some types of cancers. However, PDT-induced tumor hypoxia as a result of oxygen consumption and vascular damage can reduce the efficacy of this therapy. Measuring and monitoring intrinsic and PDT-induced tumor hypoxia *in vivo* during PDT is of high interest for prognostic and treatment evaluation. In the present study, static and dynamic ^18^F-FMISO PET were performed with mice bearing either U87MG or MDA-MB-435 tumor xenografts immediately before and after PDT at different time points. Significant difference in tumor hypoxia in response to PDT over time was found between the U87MG and MDA-MB-435 tumors in both static and dynamic PET. Dynamic PET with pharmacokinetics modeling further monitored the kinetics of ^18^F-FMISO retention to hypoxic sites after treatment. The *K*_i_ and *k*_3_ parametric analysis provided information on tumor hypoxia by distinction of the specific tracer retention in hypoxic sites from its non-specific distribution in tumor. Dynamic ^18^F-FMISO PET with pharmacokinetics modeling, complementary to static PET analysis, provides a potential imaging tool for more detailed and more accurate quantification of tumor hypoxia during PDT.

Photodynamic therapy (PDT) is a treatment modality utilizing light responsive compounds called photosensitizers which upon light irradiation generate cytotoxic singlet ^1^O_2_ from molecular oxygen found within the tumor cells and their microenvironment^1^. Because photochemistry of PDT depends on the presence of oxygen and tissue oxygen is consumed through photochemical reactions, hypoxia is then a response during PDT. Hypoxic tumor regions may be present at time of treatment due to exhaustion of blood supply through rapid tumor growth, or they might be created during PDT light treatment through rapid shut-down of tumor circulation^2^,^3^. Considering the oxygen dependence of photodynamic effect, it is conceivable that tumor hypoxia might similarly limit the effectiveness of PDT^4^. The assessment of the degree of tumor hypoxia, whether intrinsic or therapy-induced, as well as its relationship to tumor response to PDT may be of immediate relevance to the clinical application of PDT for solid tumor treatment^5^. There is a definite need to measure tumor hypoxia during treatment.

Several methods exist to measure hypoxia directly or indirectly. The “gold standard” uses polarographic electrode needles that allow direct measure of the partial oxygen pressure (pO_2_)^6^. This method, however, is invasive and can only be used in superficial tumors. Molecular imaging techniques have therefore attracted increasing interest as they are non-invasive and allow three-dimensional (3D) measurements of biological processes *in vivo*. Positron emission tomography (PET) plays an essential role in non-invasively characterizing tumor sub-types and evaluating drug target expression^7^,^8^. Radiolabeled tracers that accumulate in hypoxic tissue are used for non-invasive PET based hypoxia imaging^9^,^10^. Many of the hypoxia PET imaging tracers were ^18^F-labeled nitroimidazole compounds. Among them, ^18^F-fluoromisonidazole (^18^F-FMISO), is reduced in the absence of oxygen and retained in viable hypoxic cells. Retention of reduced ^18^F-FMISO in tissues correlates with the severity of hypoxia. Therefore, ^18^F-FMISO has been used in clinical and pre-clinical studies to provide spatially resolved images to localize and quantify tissue hypoxia^6^,^11^,^12^,^13^.

Single time point static PET imaging has been generally used in ^18^F-FMISO hypoxia imaging to quantify the total tracer uptake present in a given tissue^8^,^11^,^13^. It reveals tissue hypoxia at molecular level *in vivo* with high sensitivity and specificity^13^. Static PET scan can be performed sequentially at different time points along treatment and mean hypoxia uptake within each time point will be extracted in order to monitor therapy^14^. Besides static PET, dynamic PET with ^18^F-FMISO^15^ has also been used to assess tumor hypoxia *in vivo*. It reveals the dynamic process of tracer uptake over time and the associated kinetics such as non-specific or specific internalization of tracer in tissue. Recent pharmacokinetic studies have shown that the pharmacokinetic rate and tumor uptake of ^18^F-FMISO may vary among different tumor models^15^,^16^. Besides the hypoxia uptake, static PET imaging may reveal uptake caused by other factors such as temporary high blood flow^14^. Thus static PET alone will not be able to fully and accurately characterize tissue hypoxia of different tumor types^15^. To assess the effect of tumor hypoxia in response to PDT treatment, in the present study, ^18^F-FMISO PET was used to monitor the tumor hypoxia change following PDT in both U87MG and MDA-MB-435 xenograft models. Static and dynamic PET scans were performed and the latter have been analyzed with compartmental pharmacokinetic modeling to better quantitate tumor hypoxia before and after PDT in different tumor models.

## Materials and Methods

### Radiosynthesis of ^18^F-FMISO

The radiochemical synthesis was conducted automatically using the GE FX-FN synthesis system and modification of previously published procedures^17^. ^18^F-F^-^ was obtained from the National Institutes of Health (NIH) Clinical Center (CC) cyclotron facility from irradiation of an ^18^O-water target by the ^18^O(*p*, *n*)^18^F nuclear reaction. 100–150 mCi of ^18^F/H_2_^18^O was loaded onto an anion exchange column, dried, eluted with 1.5 mg of K_2_CO_3_ and 7.50 mg of Kryptofix 2.2.2 in 0.7 mL of CH_3_CN:H_2_O (85:15), and transferred to the reactor vial. Then, azeotropic removal of water and acetonitrile was achieved by heating the reactor to 95 °C under a stream of argon for 4 min and under vacuum for another 3 min. The dried K^18^F•Kryptofix 2.2.2 complex was then dissolved in 600 μL anhydrous DMSO containing 5–6 mg of the tosylate precursor. The reactor was sealed and heated to 110 °C for 10 min. Then, the reactor was cooled to 45 °C followed by addition of 1 mL of HCl (1 M). The temperature was increased again to 116 °C for 5 min. Thereafter, the reactor was cooled to 45 °C and the reaction solution neutralized with 1.5 mL of NaOH (0.4 M) followed by addition of 1 mL 8% ethanol/H_2_O. The diluted crude mixture was filtered through 0.45 μm filter and the product purified by a semi-preparative reversed phase HPLC (column: Phenomenex Luna 250 × 4.6 mm, 5 μm; eluent: 8% ethanol/H_2_O; flow: 4 mL/min). ^18^F-FMISO eluted at 9.5 min. The radiochemical yield of the synthesis was 23% ± 3 (n = 6).

### Cell lines and animal models

The MDA-MB-435 and U87MG cell lines were obtained from American Type Culture Collection (ATCC, Manassas, VA). The MDA-MB-435 cells were cultured in Leibovitz’s L-15 medium supplemented with 10% FBS (GIBCO, Grand Island, NY) at 37 °C and 5% CO_2_ whereas the U87MG cells were cultured and passaged in Eagle’s minimal essential medium supplemented with 10% FBS at 37 °C and 5% CO_2_.

Athymic nude mice were obtained from Harlan laboratories (Frederick, MD, USA). All experiments with live animals were conducted under protocols approved by the National Institutes of Health Clinical Center Animal Care and Use Committee (NIH CC/ACUC). The methods were carried out in accordance with the approved guidelines. The MDA-MB-435 and U87MG tumor models were generated by subcutaneous injection of 5 × 10^6^ cells in l00 μl of PBS into the right shoulder of nude mice. The mice were used for imaging and photodynamic therapy when the tumor volume reached around 100 mm^3^ (10–15 days for U87MG and 15–20 days for MDA-MB-435).

### PDT treatment

When the tumor size reached ~100 mm^3^, mice received an intravenous injection of 2-[1-hexyloxyethyl]-2-devinyl pyropheophorbide-alpha (HPPH)^18^ (0.47 μmol/kg). At 24 h post-injection, mice were anesthetized by inhalation of isoflurane (1% in 1 L/min oxygen). Each mouse was exposed to laser irradiation at a wavelength of 671 nm (Laserglow Technologies, ON, Canada) with a fluence of 75 mW/cm^2^ for 30 min^19^. Laser energy was measured by a LPE-18 laser energy meter (Coherent Portland, OR, USA). During PDT treatment process, mice were positioned in a specially-designed mouse holder, and were anesthetized by inhalation of isoflurane (1% in 1 L/min oxygen).

### Static PET imaging

Static PET imaging was performed with an Inveon^®^ PET scanner (Siemens, USA) to measure the ^18^F-FMISO tumor uptake before and after the PDT treatment. Approximately 3.8 MBq of ^18^F-FMISO was injected intravenously to tumor-bearing mice. Ten-minute static PET scan was performed at 2 h post-injection. The mice were anesthetized by inhalation of isoflurane (1% in 1 L/min oxygen) during the 10-min scan. The static PET acquisition was performed before, 1 h and 24 h after the PDT treatment. PET images were reconstructed using 3D ordered-subsets expectation maximum followed by maximum *a posteriori* algorithm with a smoothing parameter of 0.1 (OSEM-3D-MAP). The tumor region of interest (ROI) was defined by applying a threshold on the reconstructed PET images to eliminate tumor necrotic region. The mean radioactivity of the ^18^F-FMISO tumor uptake was calculated with decay correction from the entire tumor region of interest (ROI), and was compared before and after PDT treatment.

### Dynamic PET imaging

Two-hour dynamic PET imaging was performed with an Inveon^®^ PET scanner (Siemens, USA) before and 1 h, 6 h, 24 h after PDT treatment. Because the circulation time of ^18^F-FMISO was relatively long compared to other tracers with active transport mechanism (*e.g*. FDG)^15^,^20^, the acquisition time for the dynamic imaging was set to 2 h. For each dynamic scan, approximately 4 MBq ^18^F-FMISO was injected intravenously into the mice at the start of PET acquisition. Mice were anesthetized by inhalation of isoflurane (1% in 1 L/min oxygen) during the 2 h scan. Same group of mice were used for 1 h and 24 h post PDT, while a separate group of mice were used for the 6 h post PDT time point in order to avoid the residue of radioactivity remained from the 1 h post treatment group. Dynamic PET images were reconstructed into 52 time frames using (OSEM-3D-MAP). Frame rates were: 10 × 30 s, 10 × 60 s, 10 × 120 s, 10 × 150 s, 12 × 300 s.

### Pharmacokinetic modeling

The pharmacokinetic modeling^15^,^21^,^22^ was conducted to analyze the time series data from the dynamic PET imaging. To extract the tumor TAC from the PET data, the last frame of the dynamic PET image was used to define the tumor ROI. A threshold was applied to eliminate the necrotic region. The defined ROI was then applied to all time frames. Each point of the tumor TAC curve was given by the mean tracer tumor uptake within the defined ROI of each time frame. The TAC curve generated from the left ventricle ROI, which was defined with first minute frame, was used as the plasma input function.

The irreversible two-tissue compartment model^15^,^20^,^21^,^23^ was chosen as the mathematical model in the present study to analyze the ^18^F-FMISO’s transportation and internalization rate from the plasma to the tumor region. The transport of ^18^F-FMISO from vessel to hypoxia sites is considered to be purely diffusive^15^, and based on the diffusion equation^24^, the tracer’s concentration within the compartment is derived as:


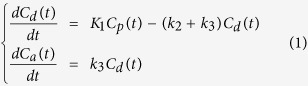


where *C*_p_(t), *C*_d_(t), *C*_a_(t) are the ^18^F-FMISO activity concentration (Bq/mL) as a function of time in the compartment corresponding to plasma, non-specific diffusive tumor area, and specific accumulative area, respectively. The plasma input function Cp(t) was the TAC curve generated from left ventricle ROI. *K*_1_ (mL/g per min) is the transport rate constant of tracer from plasma to tissue, *k*_2_ and *k*_3_ are kinetic transportation rate constants (in min^−1^) between diffusion and accumulation compartments. The activity concentration was calculated by solving the above differential equations. The signal intensity measured in a given ROI on PET images was a weighted sum of *C*_p_(t), *C*_d_(t), and *C*_a_(t), expressed as:


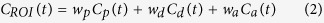


where *w*_p_ is the fractional blood volume in tumor, *w*_d_ and *w*_a_ are the relative contribution of diffusion and accumulation compartments to the rest of tumor volume (1−*w*_p_). Given the measured TAC of tumor ROI and blood input function, the *K*_1_, *k*_2_ and *k*_3_ values were calculated by fitting the measured TAC with the analytical ROI function *C*_ROI_(t) (equation (2)) using non-linear least-square regression. The goodness of the fitting was evaluated using the residual analysis^25^, and the error (between the data value and estimated function value) was limited to be less than 5%.

The compartmental modeling was conducted using a home-made Matlab object-oriented toolbox. Input parameters include plasma input TAC data, tumor ROI TAC data, and initial guess of *K*_1_, *k*_2_, *k*_3_, *w*_p_, and *w*_d_ values. According to literature^15^,^21^,^23^ and preliminary fitting test, the *K*_1_, *k*_2_, and *k*_3_ were initialized to 0.01; *w*_p_ and *w*_d_ were initialized to 0.1. The *k*_3_ values fitted from the PET data were compared before and after treatment. *K*_i_ (mL/g/min) determined in Gjedde-Patlak plot in non-compartmental graphic analysis^23^,^26^,^27^, was also calculated and compared for both tumor models at different time points.


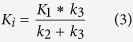


*K*_*i*_ represents the influx rate constant of the irreversibly trapping tracer from plasma to the tumor as an entire system. Thus it takes into account both specific and nonspecific distribution of ^18^F-FMISO inside tumor.

Parametric mapping^23^ of the influx rate *K*_i_ and tracer’s specific internalization rate *k*_3_ was generated using the Siemens Inveon Research Workstation. *K*_i_ mapping was applied for the whole body area using Patlak graphical analysis, with a reference tissue selected in the muscle area. The *k*_3_ map was calculated locally in the tumor area based on the irreversible compartment model presented above. Higher intensity at certain voxel of the parametric map image means higher kinetic rate value at this position. The parametric maps were also overlapped with the last frame of dynamic PET image. The tumor volume was approximated based on the ^18^F-FMISO tumor uptake of the last frame of dynamic PET. The percentage of overlapping of *K*_i_ and *k*_3_ map volume comparing to the approximated entire tumor volume was calculated and compared at different time points post PDT.

### Immunohistochemistry

Immunohistochemistry analysis was performed to observe *ex-vivo* tissue hypoxia with HydroxyprobeTM kit (Hydroxyprobe Inc., Burlington, MA). Mice bearing U87MG tumors and MDA-MB-435 tumors were injected with 60 mg/kg of pimonidazole before and 2 h after PDT treatment. After 1 h to 90 min of incubation of pimonidazole, mice were euthanized and tumors were harvested. Tissues were fixed in a 4% formaldehyde solution at room temperature overnight. Tissue sections were prepared and stained following the procedure suggested in the manufacturer’s instructions^28^. The staining slices were then visualized with ×10 magnification using Olympus BX41 microscope. All microscopic images were adjusted with the same white balance, thus tumor hypoxia was reflected directly by the pimonidazole staining color intensity (in brown color). The images were recorded with the field of view avoiding the edge of stained tumor sections. The hypoxic fraction (% of positively stained area) was quantified using ImageJ, in threshold recorded images to highlight the area positively stained by pimonidazole (in dark brown color). The threshold intensity depends on the staining intensity of each image and the results were averaged.

### Statistical analysis

Quantitative ^18^F-FMISO uptake values were expressed in %ID/g, and the kinetic parameters *k*_3_ and *K*_i_ were in min^−1^ and mL/g/min respectively. These values were presented in form of mean ± SD. The differences between two tumor models at different time points were evaluated using Student’s *t* test. *P* value less than 0.05 was considered to be statistically significant. The Pearson correlation coefficient *r* was calculated to evaluate the correlation between parameters *K*_i_ and k3, where *r* = 1 was total positive correlation and *r* = 0 was no correlation.

## Results

**Figure 1:**
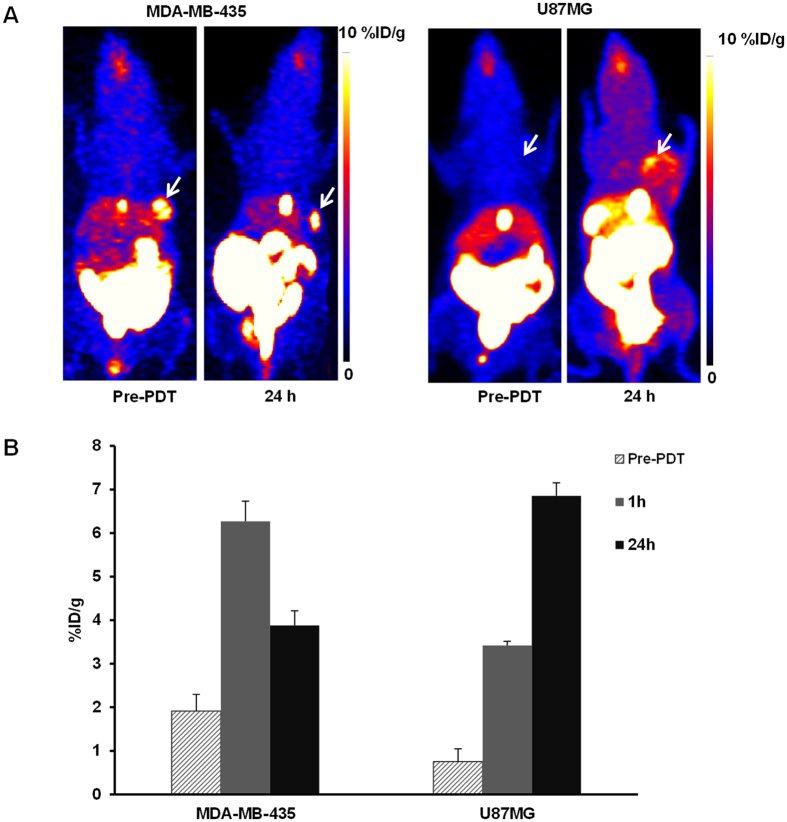
(**A**) Representative static PET 2D projection images for the MDA-MB-435 (left) and U87MG tumors (right) before and 24 h after PDT treatment. The white arrows point to the tumor area. Images were acquired 2 h post-injection of 3.8 MBq of ^18^F]FMISO, (**B**) The mean tumor uptake of ^18^F-FMISO before, 1 h and 24 h after PDT treatment for MDA-MB-435 and U87MG tumor model.

The quantitative analysis of static PET performed for both MDA-MB-435 and U87MG tumor models was presented in Fig. 1. According to Rajendran *et al*.^29^, tissue-to-blood activity ratio (T/B) higher than 1.2 indicates hypoxia in the target tissue. The baseline tumor uptake of U87MG was 0.75 ± 0.2%ID/g (n = 4) at 2 h post injection, while the heart uptake of at 2 h post-injection was at the same magnitude as the tumor uptake regardless of the inter-subject difference (mean 0.82 ± 0.1%ID/g). The T/B ratio of U87MG tumor was approximately 0.9, indicating intrinsically non-significant hypoxia in U87MG tumors. The baseline tumor uptake of MDA-MB-435 tumor was 1.92 ± 0.35%ID/g (n = 4), while much higher contrast was observed compared to the heart uptake (mean heart uptake 0.65 ± 0.3%ID/g). The T/B ratio was approximately 3, indicating intrinsic hypoxia in MDA-MB-435 tumors.

At 1 h post PDT, there was a dramatic increase in ^18^F-FMISO uptake of the MDA-MB-435 tumor (increased to 6.27 ± 0.69%ID/g, *p* = 0.0015) comparing to its baseline. However, at 24 h post treatment, the MDA-MB-435 tumor uptake decreased to 3.88 ± 0.34%ID/g. While the U87MG tumor uptake increased to 3.41 ± 0.15%ID/g at 1 h, and further increased to 6.8 ± 0.3%ID/g at 24 h post treatment. The overall increase of ^18^F-FMISO tumor uptake from baseline before treatment to that at 24 h post PDT treatment was 700% (p < 0.001) in U87MG tumor and 200% (p < 0.001) in MDA-MB-435 tumor. A clear difference in ^18^F-FMISO tumor uptake was shown on the PET images for U87MG tumor model before and 24 h after PDT, while for MDA-MB-435 tumor model this difference was less visible on PET images.

**Figure 2:**
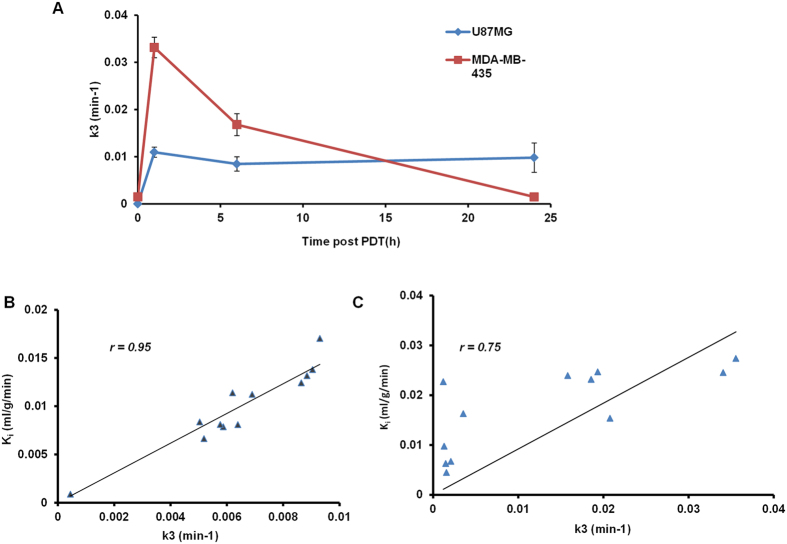
(**A**) Comparison of the mean *k*_3_ values (min^−1^) of the tumor ROI for U87MG and MDA-MB-435 tumor models, before and 1, 6, 24 h after PDT treatment. (**B**) Correlation between the kinetics parameters *K*_i_ and *k*_3_ calculated from all time points for U87MG model, with Pearson correlation coefficient r. (**C**) Correlation between the kinetics parameter *K*_i_ and *k*_3_ for MDA-MB-435 model, with Pearson correlation coefficient r.

In order to monitor the kinetics of ^18^F-FMISO retention to hypoxic sites after treatment, dynamic PET scan with kinetic modeling was performed before and after PDT. The mean value of ^18^F-FMISO kinetic rates (*K*_1_, *k*_2_, *k*_3_, *K*_i_) were analyzed for the whole tumor ROI and was compared before and after treatment (Fig. 2A, Table 1). In U87MG tumor model, ^18^F-FMISO tumor uptake intensity shown on the dynamic PET frame was nearly the same as the background intensity (<0.9%ID/g) before PDT. The reversible transportation of tracer between plasma and tumor interstitial might still exist under normoxic condition, however, the ^18^F-FMISO retention to hypoxic sites was negligible. The definition of the tumor ROI based on PET image and TAC curve extraction were not feasible. Therefore compartmental analysis was not applicable for the U87MG model before PDT treatment. To simplify analysis, the mean *k* values were considered as 0 for U87MG model before PDT. For MDA-MB-435 model, a low *k*_3_ value was observed before PDT treatment. At 1 h post treatment the *k*_3_ value of U87MG tumor increased and presented no significant change (p > 0.5) from 1 h to 24 h post treatment. The influx rate *K*_i_ of U87MG also remained consistent from 1 h to 24 h post treatment. High correlation (r = 0.9063) was found between kinetic parameters *K*_i_ and *k*_3_ calculated from different time points for U87MG (Fig. 2B). However, for MDA-MB-435 tumor model, there was a dramatic increase in *k*_3_ value at 1 h post treatment over its baseline (p < 0.001) and was three time higher than the *k*_3_ of U87MG at 1 h post treatment (p < 0.001). It gradually decreased afterwards at 6 h and 24 h time points. While the *K*_i_ value of MDA-MB-435 remained relatively constant from 1 h to 6 h and decreased at 24 h post treatment. The correlation between *K*_i_ and *k*_3_ calculated was relatively low for MDA-MB-435 tumor (r = 0.75, Fig. 2C).

**Figure 3:**
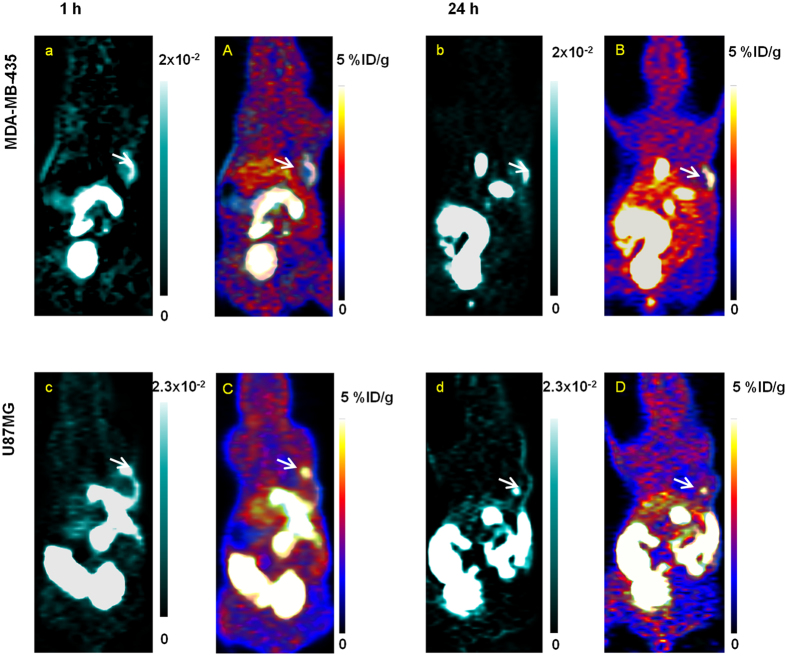
Whole body parametric mapping of *K*_i_ for U87MG and MDA-MB-435 tumor model, at 1 h and 24 h post PDT. The parametric maps of the tumor region were presented in blue and white images (a–d), and were overlapped with 2D projection of 18F-FMISO PET volumetric images (**A–D**). The scale bar next to parametric maps indicated the maximum and minimum values of *K*_i_ map. The intensity was normalized into the same scale between 1 h and 24 h for each parameter. The white arrows pointed out the location of tumor in the whole body images.

The dynamic data of the two tumor models were further analyzed at voxel level with *K*_i_ and *k*_3_ parametric maps and were compared at different time points post PDT treatment. The whole body *K*_i_ mapping was shown in Fig. 3. As *K*_i_ took into account both non-specific and specific distribution, *K*_i_ mapping was thus similar to the uptake intensity PET image. However, the *K*_i_ parametric map revealed higher (p < 0.001) tumor to tissue contrast ratio (for MDA-MB-435: 75.4 ± 8.5 at 1 h post PDT, 81.2 ± 7.8 at 24 h post PDT; for U87MG: 69 ± 15.5 at 1 h post PDT, 78.2 ± 10.8 at 24 h post PDT) than static PET image at 2 h post tracer injection (for MDA-MB-435: 3.78 ± 1.25 at 1 h post PDT, 3.25 ± 1.87 at 24 h post PDT; for U87MG: 3.25 ± 2.5 at 1 h post PDT, 3.54 ± 1.85 at 24 h post PDT) for both tumor models.

**Figure 4:**
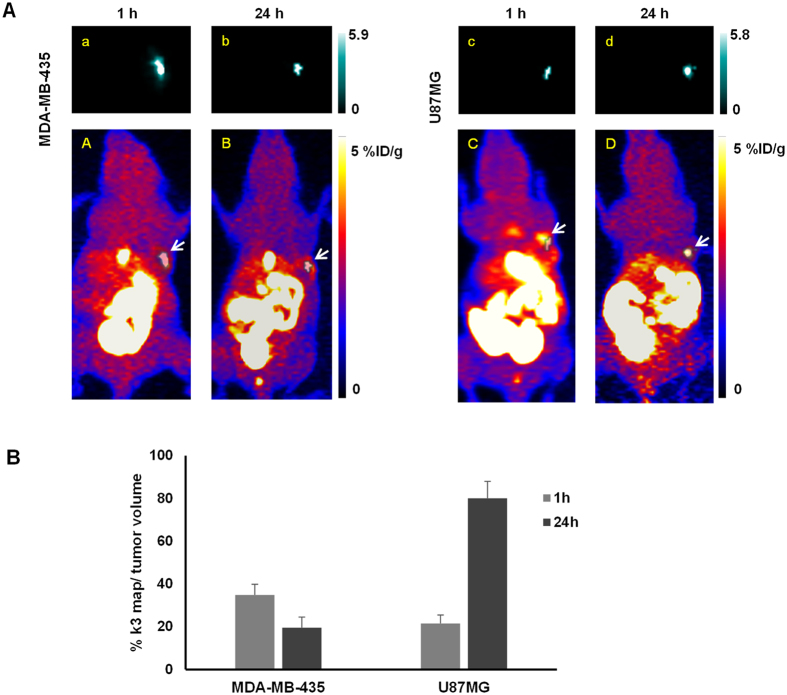
(**A**) Parametric mapping of *k*_3_ in tumor region for U87MG and MDA-MB-435 tumor model, at 1 h and 24 h post PDT. The parametric maps of the tumor region were presented in blue and white images (a–d), and were overlapped with 2D projection of 18F-FMISO PET volumetric images (**A–D**). The scale bar next to parametric maps indicated the maximum and minimum values of either *k*_3_ of each tumor model. The intensity was normalized into the same scale between 1 h and 24 h for each parameter. The white arrows pointed out the location of tumor in the whole body images. (**B**) The volume ratio of *k*_3_ map comparing to whole tumor volume at 1 h and 24 h post PDT for both tumor models.

It is interesting to compare the parametric maps of *K*_i_ and *k*_3_ around the tumor region with the tumor volume measured from PET image. For the U87MG tumor model, the *K*_i_ mapping area overlapped with approximately 95% of the entire tumor region at 1 h post PDT treatment while *k*_3_ mapping (Fig. 4) was shown only in a part of the tumor region near the tumor boundary area (approximately 21.5 ± 4% of the tumor volume, n = 4). At 24 h post PDT, the *K*_i_ mapping area overlap decreased to 80 ± 8%, however, the approximate percentage of the *k*_3_ mapping area increased to 68.5 ± 10% and was mostly located in the center of the tumor region. For the MDA-MB-435 tumor model, the *K*_i_ mapping overlapped with approximately 100% of the entire tumor region at 1 h post PDT treatment, which decreased to 75 ± 5% at 24 h post PDT. The *k*_3_ mapping covered approximately 34.8 ± 5% of tumor volume at 1 h post PDT. However, this percentage decreased to approximately 19.5 ± 5% at 24 h post PDT.

**Figure 5:**
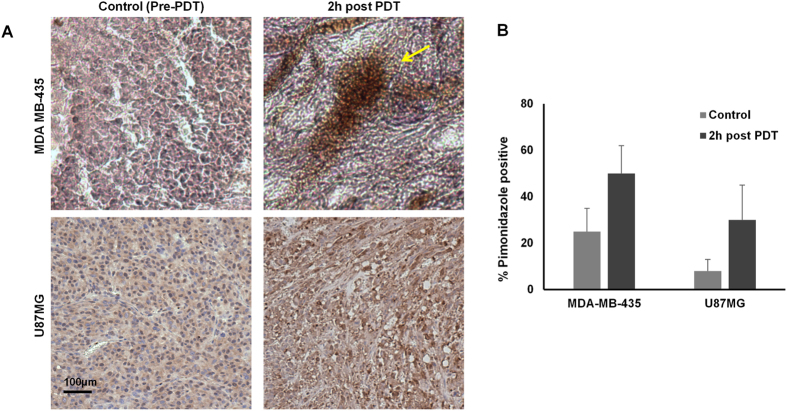
(**A**) Pimonidazole staining of U87MG and MDA-MB-435 tumor tissue, before (control) and 2 h after PDT. Hypoxic region stained by pimonidazole is shown in brown color. Yellow arrow indicates the intense stained area found in MDA-MB-435 tumor at 2 h post PDT. The images were in x10 magnification. (**B**) The hypoxic fraction (% of positively stained area) the pimonidazole staining for both tumor models.

The tumor hypoxia development in both MDA-MB-435 and U87MG tumors before and after PDT was also visualized qualitatively with pimonidazole staining (Fig. 5). The hypoxic fraction of MDA-MB-435 tumor model was 30 ± 9% *vs*. 10 ± 5% for U87MG tumor (n = 3) before PDT, indicating higher intrinsic tumor hypoxia in MDA-MB-435 tumor. At 2 h post PDT, the hypoxic fraction increased significantly in both tumor models (55 ± 12% for MDA-MB-435, p < 0.005, *vs*. 35 ± 15% for U87MG, p < 0.001). It is notable that intense brown spots were observed in the MDA-MB-435 tumor after PDT, suggesting that tumor hypoxia was increased with high heterogeneity; while in the U87MG tumor, more homogeneous enhancement was observed.

## Discussion

The responsiveness of tumor to phototherapy has been monitored previously by neovasculature imaging^30^,^31^,^32^. The oxygen consumption and tumor hypoxia during PDT has also been characterized *in vitro*^33^,^34^,^35^. Moore *et al*. assessed tumor hypoxia change in response to PDT *in vivo* using scintigraphic imaging^9^. There have also been studies of tumor hypoxia by static PET imaging^8^,^13^. However, hypoxia measurement with static PET alone may not be able to fully reflect tumor hypoxia condition^14^. In the present study, dynamic PET using hypoxic specific tracer ^18^F-FMISO combined with pharmacokinetics modeling was performed in addition to static PET.

The change of hypoxia status under PDT in two tumor xenograft models (U87MG and MDA-MB-435) was investigated in this work. The reason that these two models were chosen for comparison was that MDA-MB-435 is intrinsically hypoxic while U87MG is initially low to non-hypoxic before treatment^36^. It would be interesting to know whether different behaviors of hypoxia change would occur after PDT. Our results suggested that although U87MG tumor was initially low to non-hypoxic, therapy-induced hypoxia developed continuously subsequent to PDT (Fig. 1). On the contrary, the MDA-MB-435 tumor with intrinsic hypoxia responded quickly to PDT and produced acute tissue hypoxia at early time points post PDT as mean ^18^F-FMISO tumor uptake value increased dramatically at 1 h post PDT but then decreased at 24 h post PDT. Therefore, different patterns of tumor hypoxia variation during PDT could be observed with static PET imaging. However, the details related to these variations, e.g. whether the static tumor uptake was due to temporary increase/decrease of blood flow remained unclear. Dynamic PET with pharmacokinetics was then performed to further investigate the detailed information related to tumor hypoxia during PDT.

The *k*_3_ refers to the internalization rate of the tracer from non-specific diffusion to specific accumulation. Higher *k*_3_ value means higher specific ^18^F-FMISO internalization to hypoxic region. Although the mean tumor uptake in U87MG kept increasing (Fig. 1B), the mean *k*_3_ value remained consistent from 1 h to 24 h post PDT (Fig. 2A). The *k*_3_ parametric mapping (Fig. 4) of U87MG tumor further indicated that the percentage of ^18^F-FMISO specific internalization increased and became dominant at 24 h post PDT as tumor hypoxia developed gradually over the entire tumor region. The pimonidazole staining (Fig. 5) also showed that the tumor hypoxia developed post PDT and distributed relatively homogeneously over the staining region. This continuous development of hypoxia condition of U87MG was possibly due to its high tumor vascularity^36^ that allowed to maintain perfusion for a prolonged period of time post PDT, thus delayed the development of hypoxia due to vascular circulation shutdown. The high vessel density in U87MG model was also observed by high *K*_1_ and *k*_2_ values (Table 1), indicating a high blood supply and efflux post PDT. The influx rate constant *K*_i_ refers to tracer’s total irreversible trapping from plasma to the tumor as an entire system and it takes into account both non-specific distribution through vasculature and specific internalization of the given tracer. The strong correlation between *K*_i_ and *k*_3_ (Fig. 2B) further indicated that the ^18^F-FMISO specific trapping to hypoxic region was positively correlated with blood supply. Therefore the tumor uptake of ^18^F-FMISO and its variation in response to PDT observed in static PET were mainly related to the blood supply through tumor vasculature for U87MG tumor.

However, for MDA-MD-435 tumor model, similar to the variation of ^18^F-FMISO tumor uptake observed from static PET (Fig. 1B), the mean *k*_3_ value increased dramatically at 1 h post PDT and decreased at later time points, which corroborated with the pimonidazole result. This might be reflective of rapid induction of apoptosis and necrosis by acute tissue hypoxia^37^ at early time following PDT treatment; consequently less effective specific ^18^F-FMISO internalization was present at late time post PDT in MDA-MB-435 tumor. The dramatic decrease of *K*_i_ at 24 h (Table 1) also showed the shutdown of tracer influx through blood flow to tumor region. The relatively low *K*_1_ and *k*_2_ value (Table 1) as well as the relatively independent variation between *K*_i_ and *k*_3_ for MDA-MB-435 (Fig. 2C) indicated that ^18^F-FMISO’s specific trapping to hypoxic sites was less dependent on tumor vasculature when compared to U87MG. Therefore the variation in ^18^F-FMISO tumor uptake in response to PDT observed in static PET was not only due to the blood supply through tumor vasculature but also due to tumor intrinsic hypoxic environment for MDA-MB-435 tumor.

The parametric mapping addressed the distribution of the tracer kinetics related to tumor hypoxia at voxel level. Since the influx rate *K*_i_ referred to the kinetics rate of total tracer “trapping” into the tumor, it was expected that the whole body *K*_i_ map would show a similar pattern as the ^18^F-FMISO uptake image. However, *K*_i_ map revealed higher tumor to tissue contrast when compared to the dynamic PET image frame. The acquisition time of the last frame of 2 h dynamic PET image was 300 seconds versus 10 minutes for static PET image at 2 h post PDT. The shorter acquisition time resulted in less radioactive incidents acquired to generate one image, thus the contrast in dynamic PET scan was worse than the 10 minutes static scan. However *K*_i_ parametric mapping helped to overcome this issue by improving image contrast essentially around tumor region. It could also be beneficial for other cases where static PET images show low tumor to tissue contrast. Moreover, even though the static PET image was able to provide higher image contrast, the information presented was based on a specific time point (e.g. 10 min at 2 h post PDT) where the uptake could be contributed by a temporary radiobiological event e.g. temporal increase of blood flow^14^. While *K*_i_ and *k*_3_ maps were generated based on information of a much larger time window (the entire 2 hour post injection), there would be little to no temporal bias affecting the analysis.

The ability to map specific tracer retention in hypoxic sites with *k*_3_ parametric map and differentiate from its non-specific distribution is one of the key points that dynamic PET analysis can offer while static PET images do not. Bartlett *et al*.^38^ reported that parametric mapping especially *k*_3_ parametric map showed better correlation with the direct pO_2_ measurement than the static PET image voxel intensity. Therefore, dynamic PET with parametric mapping could potentially offer a more accurate and more detailed way than the uptake based static PET image to assess tumor hypoxia and address the heterogeneity^29^ of the tumor hypoxia during therapy at voxel level *in vivo*.

Previously, Casciari *et al*. claimed that the bioreduction product of FMISO was diffusible and was able to leave cells^12^. Bruehlmeier *et al*. also suggested the use of two-tissue reversible compartment model^39^ to assess tumor hypoxia with ^18^F-FMISO PET. The internalization of ^18^F-FMISO to hypoxic sites was considered to be reversible; the transportation rate from internalization compartment back to diffusive compartment (*k*_4_, in min^−1^) was taken into account. However, based on the PET data used in the present study, two-tissue irreversible model gave better fit than the reversible model. Table 2 showed the goodness fit evaluated by residual analysis of both irreversible and reversible compartment models applied to the dynamic PET data of MDA-MB-435 model. The fitting errors of reversible model were all greater than 5% at different time points; the *k*_4_ fit value was in the order of 10^−4^ to 10^−5^ min^−1^. This shows that the reduced or non-reduced ^18^F-FMISO that is capable of exiting from hypoxic sites is minor compared to the tracer that is constantly trapped. Considering the exchange of ^18^F-FMISO between the diffusive and accumulative compartments as a dynamic equilibrium process, the *k*_3_ in the irreversible model implemented in the present study referred to the absolute trapping rate of tracer that irreversibly stayed inside cells.

Our studies clearly indicated the need of combining both static image analysis and dynamic PET followed by parametric mapping to monitor tumor hypoxia before and after treatment. There are, however, a few concerns of using dynamic PET in practice. Dynamic PET requires relatively long acquisition time (1~2 h) with the study subject remaining anesthetized during the entire scan. There is also technical complexity from data acquisition to kinetics analysis; while static PET scan is advantageous for its simplicity and convenience. Therefore, although the approach of dynamic PET with pharmacokinetics modeling can assess tumor hypoxia more accurately following PDT than static PET, it will unlikely replace static PET in routine practice.

## Conclusion

In this study, ^18^F-FMISO PET and pharmacokinetics modeling were used to monitor tumor hypoxia before and after PDT. A clear difference in tumor hypoxia in response to PDT was observed between two tumor types: U87MG and MDA-MB-435 xenografts. In particular, dynamic PET with kinetics analysis provided information on tumor hypoxia in distinguishing the specific tracer retention to the hypoxic tumor region and the non-specific distribution in the tumor vasculature. Voxel level analysis of parametric map images allowed us to assess the heterogeneity of tumor hypoxia during PDT treatment. ^18^F-FMISO PET imaging with pharmacokinetics modeling offers a potential imaging tool to fully characterize tumor hypoxia during treatment.

## Additional Information

**How to cite this article**: Tong, X. *et al*. Monitoring Tumor Hypoxia Using ^18^F-FMISO PET and Pharmacokinetics Modeling after Photodynamic Therapy. *Sci. Rep*. **6**, 31551; doi: 10.1038/srep31551 (2016).

## Figures and Tables

**Table 1:** Kinetics parameters of U87MG tumor model and MDA-MB-435 tumor model before and after PDT at different time points.

	Pre-PDT	1 h	6 h	24 h
**U87MG (N = 4)**				
*K*_1_ (mL/g/min)	/	0.1095 ± 0.0157	0.1241 ± 0.0149	0.0763 ± 0.0063
*k*_2_ (min^−1^)	/	0.0602 ± 0.0096	0.0535 ± 0.012	0.0485 ± 0.0057
*k*_3_ (min^−1^)	/	0.0109 ± 0.0056	0.0085 ± 0.0015	0.0098 ± 0.0031
*K*_i_ (mL/g/min)	/	0.0168 ± 0.0058	0.0170 ± 0.0016	0.0128 ± 0.0022
**MDA-MB-435 (N = 4)**				
*K*_1_ (mL/g/min)	0.0115 ± 0.009	0.0255 ± 0.006	0.0264 ± 0.0086	0.0282 ± 0.003
*k*_2_ (min^−1^)	0.00123 ± 0.0004	0.00286 ± 0.00047	0.00259 ± 0.00072	0.00195 ± 0.00029
*k*_3_ (min^−1^)	0.00149 ± 0.00036	0.03314 ± 0.0027	0.01678 ± 0.0023	0.00156 ± 0.0009
*K*_i_ (mL/g/min)	0.00629 ± 0.0043	0.0235 ± 0.0054	0.0229 ± 0.0074	0.0125 ± 0.003

Values were calculated for the whole tumor region and were expressed as mean ± SD.

**Table 2:** Goodness fit (ɛ) of both irreversible and reversible two-tissue compartment models applied to dynamic PET data of MDA-MB-435 tumor model.

ɛ (%)	Pre-PDT	1 h	6 h	12 h	24 h
Irreversible	4.73 ± 1.67	4.82 ± 2.66	3.66 ± 0.78	3.89 ± 2.12	2.41 ± 1.67
Reversible	8.23 ± 3.41	10.15 ± 3.65	13.78 ± 4.21	9.54 ± 3.17	6.38 ± 1.55
